# Effects of metformin on glucose metabolism and mitochondrial function in patients with obstructive sleep apnea: A pilot randomized trial

**DOI:** 10.14814/phy2.15948

**Published:** 2024-02-12

**Authors:** Elizabeth R. M. Zunica, Elizabeth C. Heintz, Wagner S. Dantas, R. Caitlin Hebert, MaKayla Tanksley, Robbie A. Beyl, Edward C. Mader, John P. Kirwan, Christopher L. Axelrod, Prachi Singh

**Affiliations:** ^1^ Integrated Physiology and Molecular Medicine Laboratory Pennington Biomedical Research Center Baton Rouge Louisiana USA; ^2^ Translational Physiology Laboratory Pennington Biomedical Research Center Baton Rouge Louisiana USA; ^3^ Sleep and Cardiometabolic Health Laboratory Pennington Biomedical Research Center Baton Rouge Louisiana USA; ^4^ Louisiana State University Health Science Center New Orleans Louisiana USA

**Keywords:** insulin sensitivity, metformin, mitochondrial function, obstructive sleep apnea

## Abstract

Obstructive sleep apnea (OSA) is associated with increased risk for diabetes, and standard treatment with positive airway pressure (PAP) device shows inconsistent effects on glucose metabolism. Metformin is known to treat and prevent diabetes, but its effects on skeletal muscle mitochondrial function are not completely understood. Here, we evaluate the effects of metformin on glucose metabolism and skeletal muscle mitochondrial function in patients with OSA. Sixteen adults with obesity (50.9 ± 6.7 years, BMI: 36.5 ± 2.9 kg/m^2^) and moderate‐to‐severe OSA were provided with PAP treatment and randomized to 3 months of placebo (*n* = 8) or metformin (*n* = 8) treatment in a double‐blind parallel‐group design. Whole body glucose metabolism was determined by oral glucose tolerance test. A skeletal muscle biopsy was obtained to evaluate mitochondrial respiratory capacity and expression of proteins related to mitochondrial dynamics and energy metabolism. Whole body insulin‐sensitivity (Matsuda index) did not change in metformin or placebo treated groups. However, metformin treatment prevented increases in insulin release relative to placebo during follow‐up. Insulin area under the curve (AUC) and insulin to glucose AUC ratio increased in placebo but remained unchanged with metformin. Furthermore, metformin treatment improved skeletal muscle mitochondrial respiratory capacity and dynamics relative to placebo. Metformin treatment prevented the decline in whole body glucose homeostasis and skeletal muscle mitochondrial function in patients with moderate to severe OSA. Patients with OSA may benefit from the addition of metformin to prevent diabetes.

## Graphical abstract

Adjunct metformin treatment mitigates metabolic detriments in patients with obstructive sleep apnea.

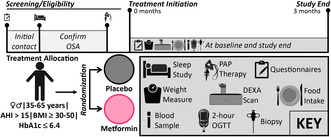

## 1 INTRODUCTION

Obstructive sleep apnea (OSA) is a prevalent sleep disorder that increases the risk of developing Type 2 diabetes (T2D) independent of obesity (Kim et al., 2021; Pamidi et al., 2010). OSA is characterized by recurrent upper airway collapse that induces intermittent hypoxia and sleep fragmentation (Qaseem et al., 2014). With time and with increasing severity, OSA causes tissue ischemia and circadian disruption leading to systemic inflammation, oxidative stress, and cytokine dysregulation, increasing the risk for metabolic diseases (Mesarwi et al., 2015). Notably, the relationship between obesity and OSA is bidirectional and feedforward. Compared to healthy weight, obesity increases the risk for OSA by 4–10.5 times (Gottlieb & Punjabi, 2020). Over time, OSA can lead to weight gain and resistance to weight loss (Kline et al., 2018; Thompson et al., 2022), ultimately increasing disease severity. Collectively, these concurrent pathophysiological circumstances promote insulin resistance, glucose intolerance, and eventually T2D (Doumit & Prasad, 2016). Currently, OSA therapy includes positive airway pressure (PAP) treatment, which resolves breathing events but modestly improves metabolic health (Kanimozhi et al., 2015; Sharma et al., 2011). As such, there is a critical need to identify treatments that improve glycemic control in patients with OSA. Importantly, while the role of OSA in diabetes related pathophysiology is well recognized, therapeutic approaches to prevent diabetes are not clinically recommended.

Metformin is one of the oldest yet most frequently prescribed medications for the treatment and prevention of T2D. Metformin was originally described as a glucose lowering agent via selective suppression of hepatic gluconeogenesis (Rena et al., 2013). Increasing evidence suggests that metformin exerts glucose lowering action in a tissue‐specific manner beyond the liver, the most prominent being changes in skeletal muscle (Galuska et al., 1994). However, the molecular mechanisms of metformin's effect on skeletal muscle remain controversial with evidence suggesting both direct and indirect benefits as well as potential risks (LaMoia & Shulman, 2020). Currently, metformin is recommended for prevention of T2D in adults with prediabetes or abnormal glycemic control (Aroda et al., 2017; *Diabetes Care*, 2022) but is not advocated in patients with OSA due to variable presentation of dysglycemia (Pamidi et al., 2010).

This pilot proof of concept study evaluated the effects of metformin on glucose metabolism and skeletal muscle mitochondrial function in non‐diabetic patients with OSA. We hypothesized that metformin treatment would improve glycemic control in OSA patients. We identified that metformin prevented decline in glucose metabolism, improved early glucose response during oral glucose tolerance test (OGTT) without changes in body weight or body composition. Mechanistically, we observed that metformin improved skeletal muscle mitochondrial function and dynamics, increasing mitochondrial content but not biogenesis. Collectively, these data indicate that in patients with OSA, metformin prevents the worsening of metabolic health and decline of skeletal muscle mitochondrial function.

## 2 METHODS

The study was conducted in conformity with the Declaration of Helsinki at Pennington Biomedical Research Center after approval from the Institutional Review Board. The study was registered at ClinicalTrials.gov (NCT04530747) before enrollment of first study participant. All participants provided written informed consent.

### 2.1 Trial design

**FIGURE 1:**
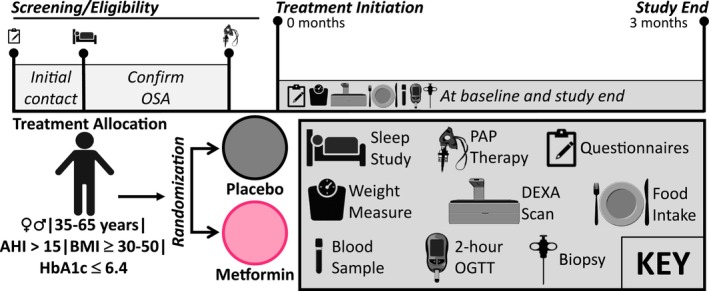
Overview of the study design. Volunteers underwent screening visits to determine eligibility, OSA severity, and prescription for PAP (positive airway pressure) therapy. Eligible participants were randomized to receive either metformin (2 g/day) or matching placebo for 3 months after baseline study measures of whole‐body glucose metabolism and skeletal muscle biopsy was obtained. Study drug was dispensed, and compliance monitored during monthly visits. At 3 months, baseline study measures were repeated, and study terminated.

This parallel randomized double‐blind placebo‐controlled trial investigated the effects of metformin therapy on glucose metabolism and skeletal muscle mitochondrial function in patients with OSA receiving standard automatic positive airway pressure (APAP) therapy (Figure 1). After determining eligibility, a study coordinator enrolled the participants, and baseline study measures were obtained. Participants were randomized by a statistician to receive either metformin or placebo (1:1) for 3 months by dynamic allocation based on age and OSA severity (AHI index score). Allocation information was provided to the research pharmacist and both participants and investigators involved with study assessment were blinded to group assignment. All participants were also provided standard OSA therapy via auto‐PAP (APAP) device and were instructed to maintain their habitual diet and physical activity during the study period to promote weight maintenance. Diet and physical activity were monitored via 3‐day food records and International Physical Activity Questionnaire (IPAQ), respectively. All baseline study measures were repeated at the end of the 3‐month study period.

### 2.2 Participants

Participants were recruited from the Greater Baton Rouge area between January 2021 and June 2021. Inclusion criteria were as follows: age 35–65 years; BMI ≥30–50 kg/m^2^; absence of overt medical or psychiatric diseases, oxygen desaturation index (ODI) ≥15 events/h; and apnea hypopnea index (AHI) ≥15 events/h. Participants with HbA_1C_ ≥6.4%, severe uncontrolled hypertension, impaired renal function (eGFR <60 mL/min/1.73m^2^), known hypersensitivity to metformin, and those taking glucose lowering, weight loss, or other medications known to affect adipose tissue and skeletal muscle metabolism, such as statins or renin‐angiotensin system targeting drugs, were excluded. Women were not pregnant, nursing, or planning a pregnancy during the 4‐month study timeframe.

**FIGURE 2:**
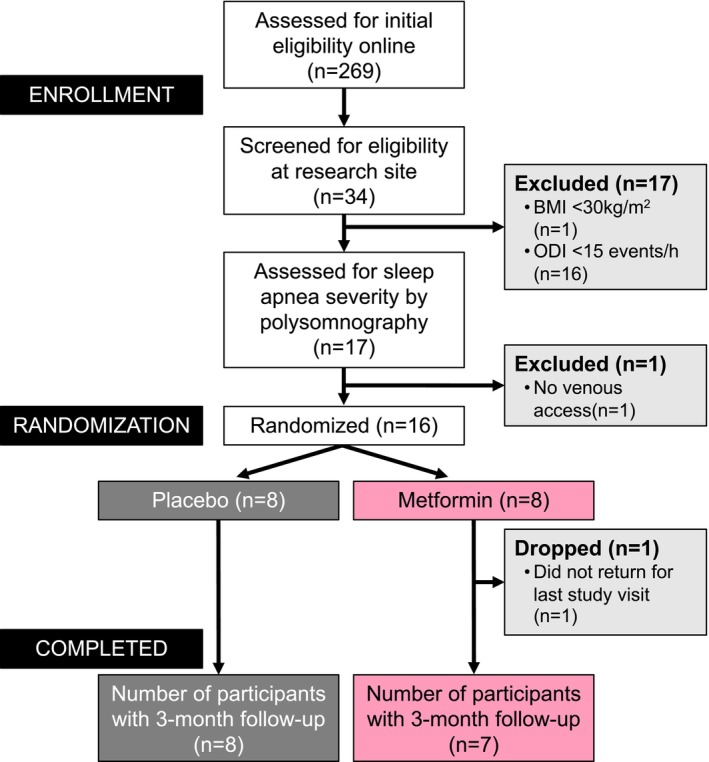
Participant flow diagram. Volunteers were initially assessed via an online questionnaire followed by an in‐person screening visit one. At this visit, participants were objectively screened for sleep apnea severity by overnight oximetry. If eligible, participants underwent an overnight sleep study to determine apnea‐hypopnea index (AHI). Randomization was 1:1 to receive either metformin or placebo by dynamic allocation based on age and OSA severity determined by AHI.

After a prescreening interview, eligibility criteria was verified during two screening visits (Figure 2). During the first visit, a medical history and physical examination was conducted along with a fasting blood draw, overnight oximetry, and determination of body weight, height, and blood pressure. Participants with ODI ≥15 events/h underwent an overnight polysomnography to determine AHI and obtain an APAP prescription. Eligible participants were scheduled for the baseline assessment visit, which was repeated after 3 months of metformin/placebo treatment. For assessments completed at study end, participants took the metformin/placebo the night before the visit at the start of the 10 h fasting.

### 2.3 Intervention and compliance

This study compared 3 months of 2000 mg extended‐release metformin versus placebo using visually indistinguishable 500 mg study drug capsules. To improve tolerance, the capsule dosage for both placebo and metformin were slowly increased over the first 4 weeks to achieve 2000 mg during Week 4 and onwards (Week 1, 500 mg [1 capsule qPM]; Week 2, 1000 mg [2 capsules qPM]; Week 3, 1500 mg [3 capsules qPM]; Week 4, 2000 mg [4 capsules qPM]). Participants taking >80% drug as determined by pill count were considered compliant. Compliance was monitored during monthly study drug dispensing visit. In addition, APAP were provided to all participants, and compliance to APAP therapy was monitored weekly via cloud‐based platform. Participants using APAP >4 h/night were considered compliant. Study follow‐up was November 2021. No side‐effects were reported in either group.

### 2.4 Study procedures

#### 2.4.1 Weight and body composition

Body weight was measured (Scale Tronix 5200, Welch Allyn, Inc; Skaneateles Falls, NY) in a fasted state while wearing a pre‐weighed gown. Body composition was measured using dual x‐ray absorptiometry (lunar iDXA, General Electric, Milwaukee, WI).

#### 2.4.2 Questionnaires

Participants completed questionnaires related to self‐perceived sleep quality (Pittsburgh Sleep Quality Index), daytime sleepiness (Epworth Sleepiness Scale), physical activity (IPAQ), and 3‐day food records.

#### 2.4.3 Oral glucose tolerance test

A 2‐h OGTT was performed using 75 g of glucose after 10 h of fasting. An intravenous line was placed, and one baseline sample was drawn at −5 min. The participant then consumed a 75 g glucose beverage within 5 min. Blood samples were drawn at 30, 60, 90, and 120 min to measure glucose and insulin. Insulin sensitivity was determined using the Matsuda index as follows: $\mathrm{ISI}\left(\text{MATSUDA}\right)=\frac{1000}{\sqrt{G_{0}I_{0}G_{\text{MEAN}}I_{\text{MEAN}}}}$ where G0 = fasting plasma glucose (mg/dL), I0 = fasting plasma insulin (mU/L), G_MEAN_ = average plasma glucose across GTT (mg/dL), and I_MEAN_ = average plasma insulin across GTT (mU/L) (Matsuda & DeFronzo, 1999). Insulin resistance was also measured using the homeostatic model (HOMA‐IR) as follows: $\frac{I_{\text{FASTING}}G_{\text{FASTING}}}{22.5}$ where I_FASTING_ = fasting plasma insulin (μU/mL) and G_FASTING_ = fasting plasma glucose (nmol/L) (Matthews et al., 1985). Glycemic control was estimated by comparing changes in area under the curve (AUC) using the Wolever method and the insulin to glucose ratio as described previously (Wolever, 2004). Insulinogenic index was calculated as follows: $\frac{{\Delta I}_{0-30}}{{\Delta G}_{0-30}}$ where ΔI_0–30_ and ΔG_0–30_ are the absolute change in insulin and glucose from 0 to 30 min, respectively. Early phase insulin was calculated as follows: ${\Delta I}_{0-30}$ where Δ*I*_0–30_ is the absolute change in insulin from 0 to 30 min. The oral disposition index was calculated as follows: IIxMI where II is the Insulinogenic Index and MI is the Matsuda Index.

#### 2.4.4 Blood chemistry

Glucose sampled from the OGTT was determined using whole blood on a Beckman Coulter chemistry analyzer system (DXC 600 Pro). Serum insulin was determined by immunoassay (Immulite; Siemens Healthcare). HbA_1c_, lipids, and renal function were determined on an automated diagnostic platform.

#### 2.4.5 Muscle tissue procurement

Skeletal muscle specimens were obtained from the medial *vastus lateralis* using a modified Bergström biopsy technique (~10 h from start of fast and administration of placebo/metformin) (Evans et al., 1982). Samples were quickly dissected of fat and connective tissue and immediately placed into preservation solution for mitochondrial respirometry studies or snap frozen in liquid nitrogen for protein studies. All muscle samples were then stored at −80 C for batched analysis. All available tissue samples were used for analysis.

#### 2.4.6 Skeletal muscle mitochondrial function

Oxidative phosphorylation (OXPHOS) and electron transfer (ET) capacity was determined by high‐resolution respirometry ex‐vivo in skeletal muscle tissue homogenates. At the time of biopsy, 30–45 mg of muscle tissue was procured and immediately placed into a biopsy preservation solution (BIOPS) for up to 4 h. as previously described (Axelrod et al., 2021). To accurately calculate the tissue homogenate concentration, a tissue homogenate preparation protocol (Zunica et al., 2021) was adapted to skeletal muscle tissue as prepared similar to what has been optimized by Ziak et al. (Ziak et al., 2015). Briefly, tissue was transferred to a mitochondrial respiration medium, (MiR05) blotted on filter paper, and weighed. ~30 mg of tissue was transferred into a chilled glass‐on‐glass dounce homogenizer with 2 mL of MiR05 and homogenized using 8–10 strokes. The homogenate was transferred to a falcon tube and the homogenizer and pestle were washed with additional MiR05 to ensure complete transfer of sample. Non‐homogenized tissue pieces were removed from the homogenate, blotted, and weighed‐ which was subtracted from the initial wet weight to determine the final sample weight. All samples were brought up to a final concentration of 4 mg/mL using additional MiR05 and 2.25 mL were added to the Oxygraph chambers. OXPHOS and ET capacity was determined using the following concentrations of substrates, uncouplers, and inhibitors: malate (2 mM), pyruvate (2.5 mM), ADP (2.5 mM), glutamate (10 mM), succinate (10 mM), tetramethyl‐p‐phenylenediamine (TMPD, 0.5 μM), ascorbate (2 mM), carbonylcyanide‐p‐trifluoromethoxyphenylhydrazone (FCCP, 0.5 μM increment), rotenone (75 nM), antimycin A (125 nM), and sodium azide (200 mM). Oxygen flux was normalized to wet weight of total homogenized tissue (mg). Cytochrome c (10 μM) was added after the addition of glutamate to confirm mitochondrial outer membrane integrity and to ensure cytochrome c was not limiting for the measurement of each OXPHOS and ET state (Figure S1).

#### 2.4.7 Tissue preparation and Western blot analysis

Muscle homogenates were prepared as described previously (Axelrod et al., 2019). Briefly, muscle tissue was homogenized using a Polytron immersion disperser in ice‐cold Cell Extraction Buffer (Invitrogen) with protease inhibitor cocktail, 5 mM phenylmethylsulfonyl fluoride (Sigma), 1 mM sodium orthovanadate (Sigma), and Phos‐STOP (Roche Applied Sciences, Indianapolis, IN). Homogenates were centrifuged for 10 min at 14,000 × g, the supernatant decanted, and tissue lysates stored at −80 C until the time of analysis. Protein concentrations were determined by BCA assay (Pierce Biotechnology, Rockford, IL). Muscle lysate 30 μg (0.75 μg/μL) were solubilized in Laemmli sample buffer containing 5% β‐mercaptoethanol and all samples except for those prepared for the anti‐OXPHOS cocktail antibody were denatured by boiling for 5 min. Sample (40 μL) was then loaded onto 4%–20% Tris Glycine gels (Novex) and separated via sodium dodecyl sulfate polyacrylamide gel electrophoresis at 125 V for 1.5 h (Invitrogen). The gels were transferred to polyvinylidene fluoride membranes (Bio‐Rad), and blocked with 5% bovine serum albumin (BSA) in Tris‐buffered saline with 0.1% Tween‐20 (TBST) for 1 h. Membranes were then incubated overnight with anti‐OXPHOS cocktail (Abcam; catalog no. ab110411), anti‐MFN1 (Proteintech; catalog no. 13798‐1‐AP, KD/KO validated), anti‐MFN2 (Proteintech; catalog no. 12186‐1‐AP, KD/KO validated), anti‐OPA1 (Proteintech; catalog no. 27733‐1‐AP, KD/KO validated), anti‐phospho‐DRP1Ser616 (Cell Signaling Technology, CST; catalog no. 3455, validated), anti‐DRP1 (CST; catalog no. 8570), anti‐PINK1 (Abcam ab23707, validated), anti‐Parkin (CST; catalog no. 4211, validated), anti‐ PGC‐1α (Santa Cruz Biotechnology; catalog no. sc‐13067), anti‐phospho‐AMPKαThr389 (CST; catalog no. 2531, validated),  anti‐AMPKα (CST; catalog no. 2532 validated), anti‐VDAC (Proteintech; catalog no. 10866‐1‐AP, KD/KO validated), anti‐phospho‐MFFSer146 (St. John's Laboratory; catalog no. STJ194724) , anti‐MFF (CST, catalog no. 84580, validated), anti‐p62 (Proteintech; catalog no. 18420‐1‐AP, KD/KO validated), anti‐LC3 (Novus Biologicals; catalog no. NB910‐40752 KD/KO validated), and anti‐HSC70 (Santa Cruz Biotechnology; catalog no. SC‐7298, validated, used as endogenous loading control) antibodies. Membranes were washed with TBST and incubated with species‐specific horseradish peroxidase‐conjugated secondary antibodies (GE Healthcare; catalog no. NA931). Immunoreactive proteins were visualized by enhanced chemiluminescence reagent (ECL Prime; GE Healthcare) and quantified by densitometric analysis using ImageJ 4. Visualized bands reactive against an internal control were subject to quantification. Values were expressed as fold induction relative to baseline placebo normalized to loading control (HSC70). For all blots, an internal tissue sample control was used to demonstrate the protein was observed at the predicted molecular weight and for quantification across blots.

#### 2.4.8 Citrate synthase activity

Enzymatic activity of citrate synthase was determined in snap‐frozen tissue (~10 mg) using a commercially available colorimetric assay (Sigma‐Aldrich, St. Louis, MO, USA) as described previously (Axelrod et al., 2021). Briefly, 500uL of tissue homogenate was pelleted and then resuspended and further homogenized in 20 μL of Cell MT lytic buffer and incubated on ice for 10 min. Homogenates were centrifuged at 20,000 × g for 10 min at 4 C to pellet tissue debris. The supernatant was transferred to a fresh tube. Supernatant (5 μL) were added to master mix containing 1× assay buffer, 30 mM acetyl CoA, 10 mM DTNB in a 96‐well plate in triplicate. Absorbance was then measured on a plate reader set to kinetic mode (412 nm, 4 min duration, 10 s intervals) before and after the addition of 10 mM oxaloacetate. Data are expressed as μmol of activity per minute per milligram of protein.

#### 2.4.9 Statistical power

The primary goal of this pilot study was to collect preliminary data to determine the treatment effect and variability estimates to inform design of a larger definitive clinical trial. Our initial plan was to enroll 20 participants with complete data from 18 participants. However, the study had to be terminated early after enrollment of 16 participants because of the recall of APAP device being provided to our study participants and our inability to procure device from other manufacturers. Further, for safety, the participants already enrolled in the study were asked to stop using APAP and seek clinical guidance from their primary care providers. However, all participants continued taking study drug and end of study measures were obtained at 3 months. Generalizability may be limited by small sample size. Full protocol and statistical analysis plan is available on ClinicalTrials.gov. Since no prior study has examined the effect of metformin treatment in OSA patients receiving APAP therapy, a formal power analysis was not possible. The primary outcome was Matsuda index, secondary outcomes include additional OGTT data, and exploratory outcomes were the mitochondrial investigations. The outcomes were not changed after the trial commenced. Additional details related to study conduct are in Figure S1—Supplement Information.

#### 2.4.10 Statistical analysis

Within‐ and between‐group differences were assessed by linear mixed effect models with corresponding contrasts, as applicable. Significance was accepted as *p* < 0.05. Data are presented as indicated in each table and figure legend.

## 3 RESULTS

### 3.1 Metformin did not alter body composition, daytime sleepiness, or sleep quality

Baseline participant characteristics are presented in Table 1; Tables S1 and S2. All participants had at least class I obesity, with the majority having borderline Class II obesity (68.8%). Most of the study participants were male and had severe OSA (81.3%, AHI >30 events/h). Of the 16 participants, two had prediabetes as defined by HbA_1C_ >5.7. Participants were insulin resistant with an average HOMA‐IR of 4.83 ± 1.13 and 4.14 ± 1.13 at baseline for the placebo and metformin group, respectively (Table S2). No baseline differences were observed between groups. All participants were compliant to study drugs. APAP compliance was 38% and 75% in the metformin and placebo groups. Of the participants non‐compliant to APAP treatment, two were nontolerant and five were asked to stop APAP use due the safety concerns emanating from recalled devices.

**TABLE 1:** Baseline participant characteristics.

Characteristics	Placebo (*n* = 8)	Metformin (*n* = 8)	*p* value
Age (years)	51.5 ± 5.0	50.3 ± 8.3	0.72
Sex, (male)	6 (75%)	7 (87.5%)	0.52
Race, (white)	8 (100%)	7 (88%)	0.3017
Ethnicity, (non‐hispanic)	8 (100%)	7 (88%)	0.3017
Weight (kg)	107.3 ± 19.8	112.1 ± 9.1	0.54
BMI (kg/m^2^)	36.3 ± 3.5	36.6 ± 2.3	0.84
HbA_1C_ (%)	5.5 ± 0.3	5.5 ± 0.3	1
Glucose (mg/dL)	98.5 ± 12.4	97.8 ± 8.6	0.89
Insulin (μU/mL)	18.6 ± 11.3	17.3 ± 9.4	0.81
HOMA‐IR	4.8 ± 3.8	4.1 ± 2.4	0.67
MAP (mmHg)	94.8 ± 8.3	93.5 ± 8.1	0.75
SBP (clinical, mmHg)	121.8 ± 13.2	120.3 ± 13.2	0.82
DBP (clinical, mmHg)	81.4 ± 6.1	80.1 ± 7.1	0.71
Heart rate (beats/min)	65.8 ± 6.6	62.5 ± 8.3	0.4
ODI (events/h)	32.1 ± 25.0	24.8 ± 9.2	0.45
AHI (events/h)	52.1 ± 21.0	54.4 ± 22.5	0.83
Cholesterol (mg/dL)	212.5 ± 41.8	212.8 ± 44.3	0.99
HDL (mg/dL)	51.0 ± 11.0	48.8 ± 13.2	0.71
LDL (mg/dL)	133.1 ± 37.5	137.3 ± 29.0	0.81
Triglyceride (mg/dL)	141.8 ± 72.4	133.3 ± 61.5	0.81
Non HDL cholesterol (mg/dL)	274.9 ± 72.4	270.6 ± 85.5	0.92
eGFR (mL/min/1.73 m2)	87.8 ± 14.9	79.4 ± 14.6	0.28

Abbreviations: AHI, apnea hypopnea index; DBP, diastolic blood pressure; eGFR, estimated Glomerular filtration rate; HbA_1C_, glycated hemoglobin; HDL, high density lipoprotein; LDL, low density lipoproteins; MAP, Mean arterial pressure; ODI, oxygen desaturation index; SBP, systolic blood pressure.

*Note*: Data are mean and standard deviation.

As designed, after 3 months of treatment, weight, physical activity, and habitual diet remained stable in both metformin and placebo group (Table S1). No change in total body fat mass or fat‐free mass was observed in either metformin or placebo group. Neither metformin nor placebo reduced daytime sleepiness (reduction in ESS score), but tendency to improve self‐reported sleep (reduction in PSQI score) was observed in metformin treated group (Table S1).

### 3.2 Metformin improved acute phase insulin sensitivity and prevented an increase in HbA_1c_

**FIGURE 3:**
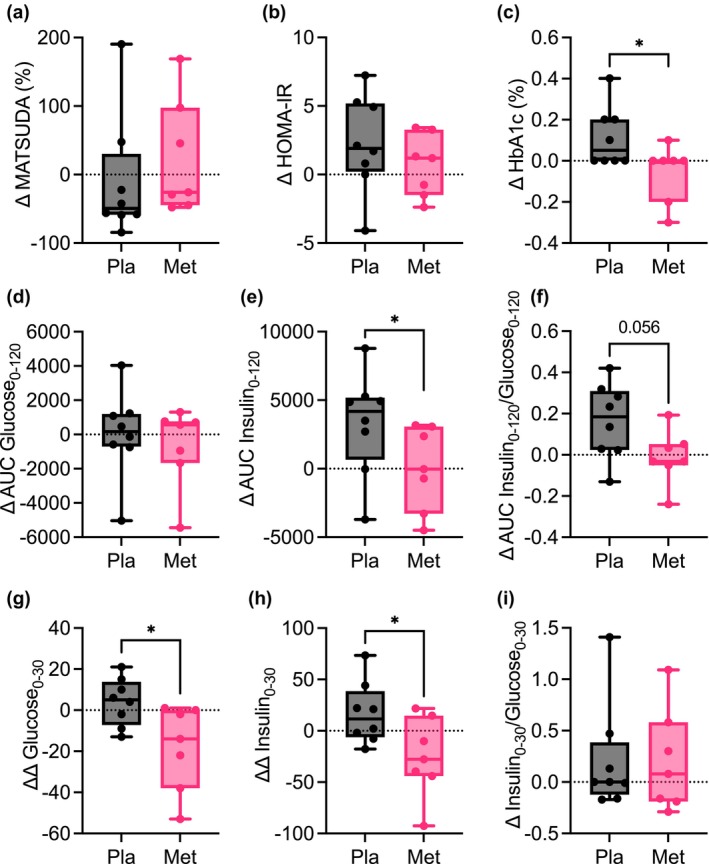
Metformin prevents decline in glucose homeostasis and glycemic control in patients with OSA. Change from baseline after 3 months of treatment with placebo or metformin in the (a) Matsuda index, (b) HOMA‐IR, (c) HbA_1c_ (d) AUC glucose, (e) AUC insulin, (f) insulin to glucose AUC ratio, (g) acute glucose response, (h) acute insulin response, and (i) acute insulin to glucose ratio (insulinogenic index). *N* = 8 placebo and *N* = 7 metformin. Data are mean and Std‐dev. *p < 0.05 between group differences determined from mixed model analysis, *p*‐value included for *p* < 0.13. HOMA‐IR: Homeostatic model assessment for insulin resistance; AUC_120_, area under the curve during 120 min of oral glucose tolerance test; AUC_30_, area under the curve during 30 min of oral glucose tolerance test.

No between‐group differences were observed in the Matsuda index, an index of whole‐body insulin sensitivity (Figure 3a). HOMA‐IR remained unchanged in metformin group but trended to increase in placebo group (Figure 3b; Table S2). There was an improvement from baseline in HBA_1c_ with metformin compared to placebo (Figure 3c; Table S2). Compared to baseline, fasting and glucose levels during OGTT remained unchanged during 3‐month follow‐up in both metformin and placebo treated groups (Table S2; Figure 3). However, insulin secretion during OGTT significantly increased during follow‐up only in placebo treated group. Consequently, relative to baseline, an increase in insulin to glucose AUC ratio was apparent in placebo treated group but did not change in metformin group (Figure 3d–f). Further, we observed that metformin improved the acute phase glucose clearance and insulin release compared to placebo (Figure 3g,h). These improvements were proportional and thus, no differences in the insulin to glucose ratios between groups were observed (Figure 3i). Overall, the placebo group demonstrated worsened glucose metabolism with increased insulin secretion to maintain glucose levels and increased HbA_1c_, independent of changes in BMI, which was ameliorated by metformin.

### 3.3 Metformin improved skeletal muscle mitochondrial function

**FIGURE 4:**
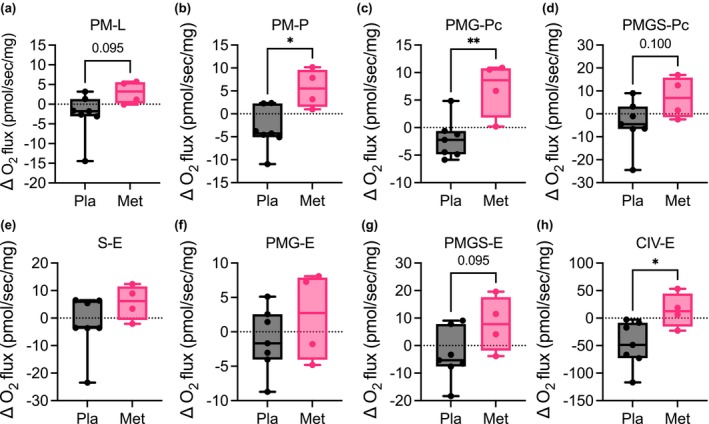
Metformin treatment improves skeletal muscle mitochondrial function in patients with OSA. Change from baseline after 3 months of treatment with placebo or metformin in (a) pyruvate + malate leak, (b) pyruvate + malate phosphorylation, (c) pyruvate + malate + glutamate + cytochrome c phosphorylation, (d) pyruvate + malate + glutamate + succinate + cytochrome c phosphorylation, (e) pyruvate + malate + glutamate related electron transfer, (f) succinate related electron transfer, (g) pyruvate + malate + glutamate + succinate related electron transfer, and (h) Complex IV related electron transfer. *N* = 7 placebo and *N* = 4 metformin. Data are mean and Std‐dev. *p < 0.05, **p < 0.01, between group differences determined from mixed model analysis. *p*‐value included for *p* < 0.13.

The tissue‐specific molecular mechanisms of metformin are not yet fully elucidated. We tested the functional capacity of mitochondrial pathways of coupling control, including NADH‐linked (N‐linked), succinate‐linked (Complex II), and Complex IV activity in skeletal muscle tissue homogenates using high resolution respirometry (Figure 4a–h; Figure S1). Metformin improved mitochondrial N‐linked (OXPHOS) and Complex IV ET capacity in skeletal muscle tissue homogenates compared to controls, indicating there was no inhibition of skeletal muscle Complex I function with metformin (Figure 4b,c,h). Overall, we found there was a decrease in mitochondrial capacity over time with the placebo, whereas there was a prevention of the decrease in mitochondrial capacity with metformin after 3 months of treatment (Figure 4a–h; Table S3). These data suggest that metformin maintains skeletal muscle mitochondrial function and does not inhibit the respiratory capacity of Complex I in skeletal muscle mitochondria in patients with obesity and moderate to severe OSA.

### 3.4 Metformin did not alter mitochondrial complex protein expression or mitochondrial biogenesis

**FIGURE 5:**
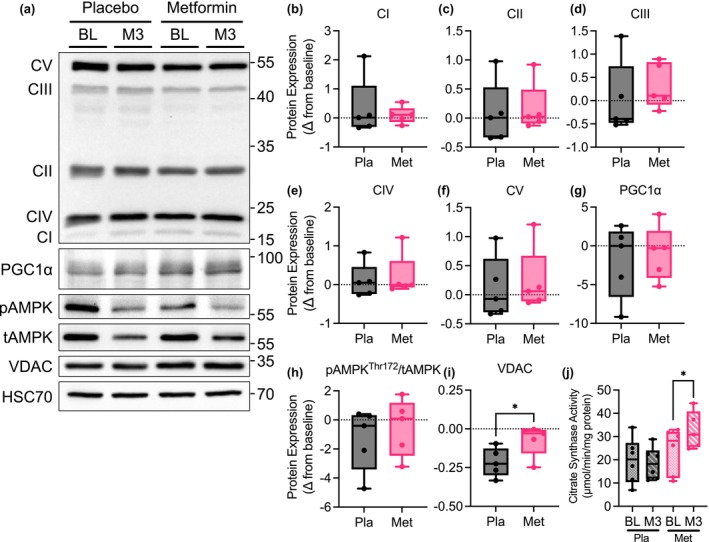
Metformin treatment does not alter skeletal muscle mitochondrial respiratory complex protein expression or biogenesis in patients with OSA. (a) Representative immunoblots of respiratory complex V (CV), III (CIII), IV (CIV), II (CII), and I (CI), PGC‐1α, pAMPK_Thr172_, total AMPK, and VDAC. (b–i) Quantification of change in protein expression from baseline to 3 months of treatment with placebo or metformin. A‐I, *N* = 5 placebo and *N* = 5 metformin. Data are mean and Std‐dev. *p < 0.05 between group differences determined from mixed model analysis. (j) Citrate synthase activity at baseline (BL) and 3‐month follow‐up (M3) in placebo and metformin treated group. *N* = 6 Placebo baseline, *N* = 6 Placebo M3, *N* = 6 Metformin baseline, and *N* = 5 Metformin M3. Data are mean and standard deviation. * *p* < 0.05 within group differences determined from mixed model analysis.

Given the changes in mitochondrial function, we interrogated the potential mechanism of the increase in capacity by measuring protein markers of the OXPHOS complexes and mitochondrial biogenesis. Metformin did not increase the total protein expression of OXPHOS CI, CII, CIII, CIV, or CV (Figure 5a–f). Additionally, there was no difference in the protein expression of the transcriptional coactivator and master regulator of mitochondrial biogenesis peroxisome proliferator‐activated receptor‐ γ coactivator‐1α (PGC‐1α) (Figure 5a,g). Consistent with a lack of inhibition of Complex I and lack of change in whole‐body insulin sensitivity, there was also no difference in AMP‐activated kinase (AMPK) phosphorylation (Figure 5a,h), the energy sensor and activator skeletal muscle mitochondrial biogenesis. We did find that 3 months of metformin attenuated the decrease observed with placebo in expression of voltage‐dependent anion channel (VDAC) (Figure 5a,i), the highly expressed outer mitochondrial membrane transporter and channel of mitochondrial metabolites, molecules, and ions. Additionally, citrate synthase activity, a biomarker of mitochondrial content (Larsen et al., 2012), increased with metformin treatment but not with placebo (Figure 5j). These data indicate that the increase in mitochondrial function with metformin may result from an increase in overall mitochondrial content but not by a change in the abundance of specific respiratory complexes. Additionally, the maintenance of VDAC expression may indicate that mitochondrial transport is protected with metformin and that the mitochondria may have improved overall quality rather than a change in quantity.

### 3.5 Metformin prevented the decline in skeletal muscle mitochondrial fusion

**FIGURE 6:**
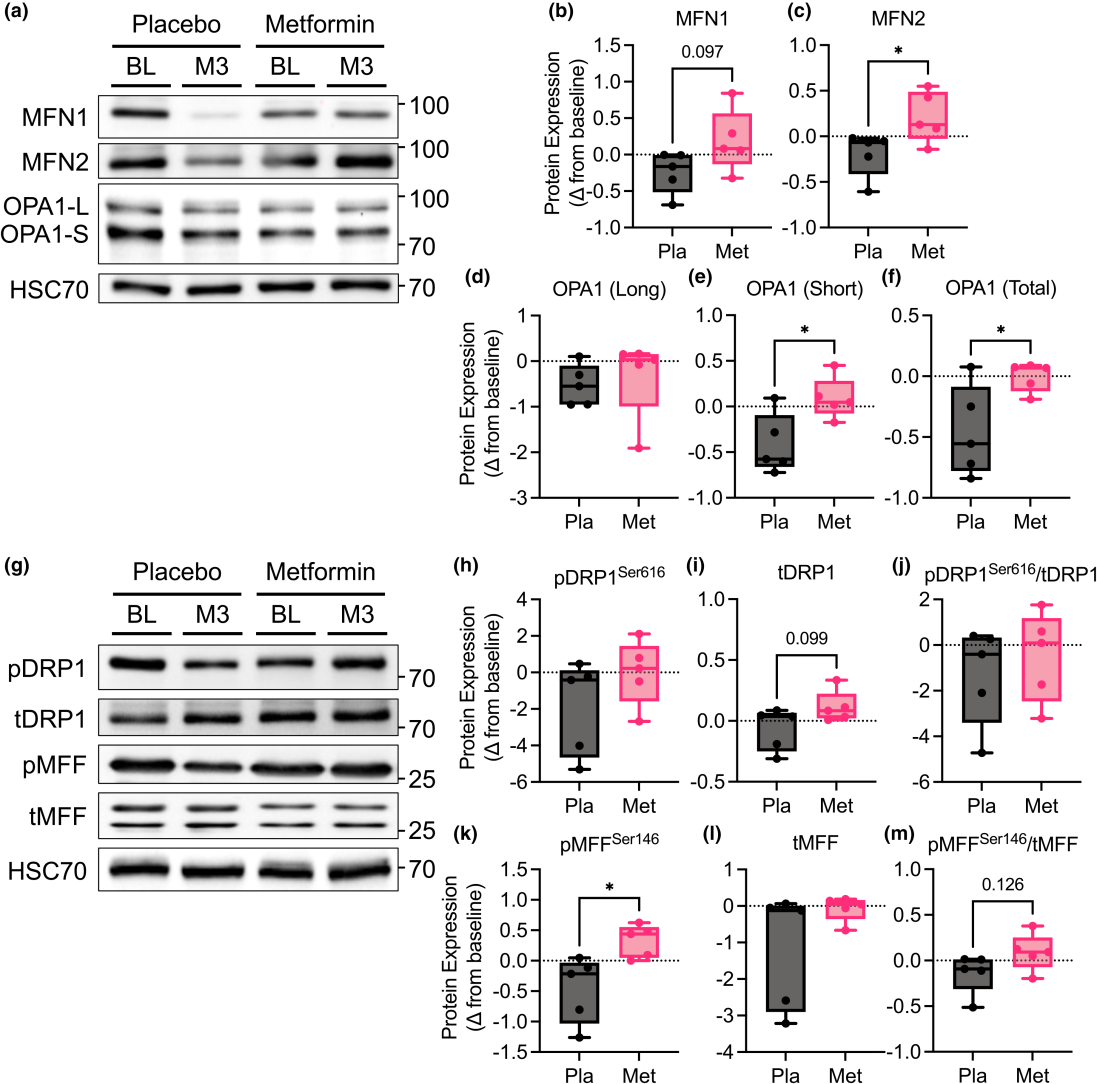
Metformin treatment prevents the decline in skeletal muscle mitochondrial dynamics in patients with OSA. (a) Representative immunoblots of MFN1, MFN2, OPA1 long (‐L), OPA1 short (‐S), and HSC70. (b–f) Quantification of change in protein expression from baseline to 3 months of treatment with placebo or metformin. (g) Representative immunoblots of pDRP1_Ser616_, total DRP1, pMFF_Ser146_, and total MFF. (h–m) Quantification of change in protein expression from baseline to 3 months of treatment with placebo or metformin. *N* = 5 placebo and *N* = 5 metformin. Data are mean and Std‐dev. * *p* < 0.05 between group differences determined from mixed model analysis, *p*‐value included for *p* < 0.13.

Mitochondria are dynamically regulated through balanced cycles of fission and fusion, yielding morphologically plastic networks that can alter function independent of changes in overall mitochondria content (Fealy et al., 2018). Since there was no indication of a change in biogenesis, we measured the effect of metformin treatment on key regulators of mitochondrial dynamics. Overall, we found a decrease from baseline in markers of both fission and fusion mediators with placebo (Figure 6a–c), consistent with decreasing mitochondrial function we observed. With metformin, there was a trending increase in the expression of outer mitochondrial membrane protein mitofusion 1 (MFN1) and an increase the expression of outer mitochondrial membrane protein mitofusion 2 (MFN2) (Figure 6a–c) compared to placebo. The expression of the long form of inner mitochondrial membrane protein, dynamin like GTPase Optic Atrophy‐1 (OPA1) was not changed, but the short form and total OPA1 decreased with placebo and thus, overall increased with metformin compared to placebo (Figure 6a,d–f). There were some indications of changes in markers of fission mediators with a trending increase in total dynamin‐1‐like protein (DRP1) and an increase in phosphorylated mitochondrial fission factor (MFF) with metformin relative to placebo, but overall, there were not differences in the ratio of phosphorylated to total protein (Figure 6g–m). These data indicate that metformin may enhance mitochondrial outer membrane fusion without largely affecting mitochondrial fission.

### 3.6 Metformin improved skeletal muscle quality control

**FIGURE 7:**
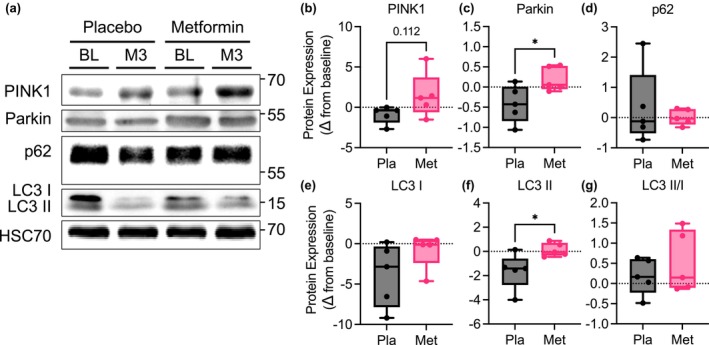
Metformin treatment prevents a decline in skeletal muscle mitophagy in patients with OSA. (a) Representative immunoblots of PINK1, Parkin, p62, LC3I, and LC3 II. (b–g) Quantification of change in protein expression from baseline to 3 months of treatment with placebo or metformin. *N* = 5 placebo and *N* = 5 metformin. Data are mean and Std‐dev. * *p* < 0.05 between group differences determined from mixed model analysis, *p*‐value included for *p* < 0.13.

With increased mitochondrial fusion, we hypothesized that metformin improved mitochondrial networks and overall quality compared to placebo. Overall, we found that markers of quality control and mitophagy were decreased from baseline with placebo (Figure 7a–g). With metformin, there was a trending increase in the expression of the mitochondrial quality control protein PTEN‐induced kinase 1 (PINK1) and in increase in Parkin compared to placebo, which was attributable to an overall decrease in Parkin from baseline to M3 with placebo, but no difference in the autophagy mediator p62 (Figure 7a–d). With metformin there was no difference in long microtubule‐associated protein 1 light chain 3 (LC3) isoform (LCI) but there was an attenuation of the decrease of LC3II, although there was no difference in the ratio of the isoforms between treatment groups (Figure 7a,e–g). These data indicate skeletal muscle mitochondrial function was preserved through retained mitochondrial networks and quality.

## 4 DISCUSSION

Obesity potentiates OSA, which has a compounding effect on overall morbidity and mortality and quality of life (Su et al., 2021). Patients with OSA are at a high risk of developing T2D given the metabolic ramifications of chronic intermittent hypoxia and sleep fragmentation. However, with a lack of clinical evidence, they are not currently advocated for to receive metformin to prevent T2D. Here, we provide proof of concept evidence that metformin can attenuate the decline in metabolic function observed in patients with moderate to severe OSA. We show that metformin attenuates deterioration of glucose metabolism, preserves skeletal muscle mitochondrial function, and prevents a decline in skeletal muscle mitochondrial quality control.

The molecular mechanisms of metformin's effect on skeletal muscle remain unclear. Strong evidence suggests indirect benefits resulting from improvements in glucose disposal. Other evidence points towards potential direct benefits or risks depending on whether the doses achieved in vitro or preclinical models are achieved in human skeletal muscle. For example, high‐dose metformin treatment suppresses mitochondrial Complex I activity (El‐Mir et al., 2000; Wessels et al., 2014), and thus could compromise muscle oxidative capacity. In muscle cells and skeletal muscle tissue, high doses of metformin have been shown to inhibit mitochondrial Complex I and that Complex I inhibition and subsequent adenosine monophosphate‐activated protein kinase (AMPK) activation are required to elicit metformin's insulin sensitizing effect (Pavlovic et al., 2022; Wessels et al., 2014). Others have demonstrated that metformin's glucose lowering effect is primarily a result of inhibition of hepatic gluconeogenesis (LaMoia & Shulman, 2020) and shown to be independent of AMPK activation (Foretz et al., 2010; Rena et al., 2017) Furthermore, the effects of metformin on muscle are secondary to the hepatic mechanism and improvement of overall insulin sensitivity. However, it is likely that Complex I inhibition in the muscle may not occur in doses used clinically for anti‐diabetic effects (LaMoia & Shulman, 2020). Additionally, it is unclear if the effects of metformin on skeletal muscle mitochondria require improvement in whole‐body insulin sensitivity. We observed a decrease in metabolic function as well as in skeletal muscle mitochondrial function over the course of 3 months in patients with OSA receiving the placebo control. Conversely, we found that metformin treatment prevented the decline compared to placebo. A difference in early but not late‐phase glucose response was detected, and thus, there was no overall change in whole‐body insulin sensitivity between groups even though metformin protected against an increase in HbA_1c_. It is possible that with a longer study duration, increased study sample size or a more sensitive test, a difference in insulin sensitivity could be detected between groups. Alternatively, given that this study population did not have diabetes at baseline, the main metabolic benefit metformin may offer is lower overall glucose and fasting glucose and the first‐phase glucose clearance and insulin response. Interestingly, despite similar insulin sensitivities based on the Matsuda Index, metformin protected against a decline skeletal muscle mitochondrial function. These data suggest that in patients with OSA, declines in tissue level metabolic functions may precede declines in tissue and whole‐body insulin sensitivity even with weight stability and that metformin may prevent this decline.

In skeletal muscle, we found an overall decline in mitochondrial Complex IV function in placebo and a protection from this decline with metformin. Notably, this defect occurred upstream of Complex IV, with a significant decrease in N‐linked OXPHOS but intact ET capacity. These findings indicate that OXPHOS of N‐linked substrates was restricted in placebo but not metformin. We have recently demonstrated that mitochondrial respiratory function and ultrastructure remain intact despite lipid‐induced insulin resistance (Axelrod et al., 2021), indicating that the decline observed in the OSA population may not result from worsened glucose handling and is likely derived from other OSA‐related derangements such as obesity, intermittent hypoxia, sleep fragmentation, and inflammation. Further investigations into the differences in ADP sensitivity seen between the groups will add insight into whether the kinetics are altered and if there are differences in ADP sensitivity at physiological ranges as well.

Mechanistically, we attributed preservation of skeletal muscle mitochondrial function to retention of quality control regulation. We did not find a change overall in mitochondrial biogenesis, but rather a retention of mitochondrial fusion and networking. Interestingly, we found increases in MFF phosphorylation, PINK1, and Parkin, and LC3‐II independent of AMPK activation. LC3‐I is localized to the cytosol and after conjugation is activated to the LC3‐II isoform to induce autophagy. Since LC3‐II is present on both inner and outer autophagosome membranes it can be degraded inside the autolysosomes and inhibition of lysosomal proteases can prevent the degradation of LC3‐II, without affecting LC3‐I (Mizushima & Yoshimori, 2007). Given this dynamic regulation of LC3‐II, the increase in LC3‐II with metformin may be an indication of upregulated autophagosome formation, suggesting improved mitochondrial turnover. With the lack of increased AMPK and DRP1 phosphorylation it is possible that the efficiency of mitophagy is altered, possibly at the level of autophagic degradation or lysosomal function rather than an increase in total mitophagy flux. Further study of the mitophagy flux and lysosomal machinery are warranted.

Our data demonstrate that AMPK activation and changes in whole body insulin sensitivity were not observed at the timepoint when metformin‐induced changes in skeletal muscle mitochondrial capacity were detected in patients with OSA and obesity. Given the decrease in VDAC expression and in Complex IV capacity in placebo without a change in AMPK activation, it is possible that ATP transport out of the mitochondria remains proportional to the efficiency of ATP production such that the ADP/ATP ratio is not significantly altered. With metformin, demand for ATP may be retained from baseline and the mitochondrial capacity to produce ATP and transport ATP remain intact without changing the overall ADP/ATP ratio, thus yielding an AMPK‐independent mechanisms of metformin on skeletal muscle mitochondria. These data indicate that metformin may protect against a decline in skeletal muscle ATP demand and mitochondrial function over time in patients with OSA.

Our study has both strengths and limitations. Key strengths include the target population of newly diagnosed patients with moderate to severe OSA confirmed by laboratory based overnight sleep‐study. Accommodations were made to increase accessibility of the trial and comparisons between groups, for example providing an APAP machine for all participants. We also performed specialized high resolution respirometry of skeletal muscle biopsies to directly measure mitochondrial functional capacity. Several strategies, including isolated mitochondria, chemical permeabilization of fibers, and tissue homogenates can be employed to examine mitochondrial function all with advantages and limitations. Permeabilized human skeletal muscle fibers are commonly employed for respirometry due to small sample requirements (<10 mg) and high recovery of mitochondria from tissue compared to isolated mitochondria which require 40–60 mg of starting tissue and loss of up to 80% of mitochondria (Picard et al., 2011). Like permeabilized muscle fibers, using skeletal muscle homogenates has the advantage of requiring a small sample amount with high retention of mitochondrial content. Unlike muscle fibers, experiments with homogenates can be conducted at low oxygen concentrations, limiting the effects of diffusion and ultimately improving sensitivity. Additionally, homogenates may be advantageous when muscle bundle integrity is not optimal due to tissue fragility. Chemical permeabilization of fiber bundles is generally less damaging to the outer mitochondrial membrane than mechanical homogenization and as a result, homogenates typically have higher rates of cytochrome c release (Larsen et al., 2014). For this reason, cytochrome c is supplemented into the chamber to ensure it is not limiting to respiration, which produces largely comparable rates to permeabilized fibers (Jiroutkova et al., 2015) (Ziak et al., 2015). Monitoring the change in respiration after the addition of cytochrome c is often used to measure membrane integrity, but more studies are needed to validate commonly used thresholds across preparations and disease states (Kuang et al., 2022; Perry et al., 2013). In this study, homogenates were selected over permeabilized fibers to address the low tissue yield, fiber bundle quality from the patient population, and ability to limit oxygen dependence. However, whether these results would be replicated in permeabilized fibers is unclear.

Additional limitations included a small sample size, short duration of treatment, exclusion of patients with overt abnormalities in glucose homeostasis, and insensitivity of the OGTT to detect small changes in insulin sensitivity. This study used metformin extended‐release which has a longer half‐life and lower peak drug concentration than metformin immediate release, limiting side‐effects and improving tolerability. Of note, the muscle biopsy was obtained about 10 h from last medication dose, thus, muscle related outcomes may be capturing some acute effects of the medication which cannot be fully parsed out from the chronic effects. Additionally, we were unable to assess the effects of APAP treatment in our study as we had to ask five out of 16 participants to stop using APAP due to safety concerns. The use of APAP likely reduces hypoxia resulting in strong physiological consequences and improves sleep as evident by improvement in PSQI score. However, our study did not measure sleep at the follow‐up period. Nevertheless, most of our placebo randomized participants (six out of eight) used APAP as directed and a sensitivity analysis only including APAP compliant participants in placebo group did not change the directionality of the outcomes. Conversely, only two of the eight participants in metformin group used APAP. Notably, more participants in the placebo group used APAP and presumably have improved sleep, but they continued to have a worsening of glucose metabolism at the 3‐month follow‐up period. On the other hand, most participants in the metformin group did not use APAP and continued to have poor sleep but were able to maintain the whole‐body insulin‐sensitivity at the 3‐month follow‐up period. It is likely that metformin failed to improve insulin sensitivity from baseline because of non‐treatment of OSA.

## 5 CONCLUSION

Metformin prevents the decline in skeletal muscle respiratory function, in part, by enhancing mitochondrial quality control in patients with OSA, which preceded changes in whole body insulin action. Furthermore, these data demonstrate the risk of rapid decline in mitochondrial function and insulin sensitivity in patients with moderate to severe OSA. Clinical trials of longer duration and adequate sample size are warranted to definitively determine the beneficial effects of metformin in improving glucose metabolism in APAP treated OSA patients. Additionally, a more sensitive determination of peripheral insulin sensitivity by application of a hyperinsulinemic‐euglycemic clamp in patients with and without OSA and in response to metformin is warranted. Subsequently, the mechanism of action whereby metformin stabilizes skeletal muscle mitochondrial function and quality control in OSA remains ambiguous. As such, future studies using cellular and animal models of OSA in the context of metformin treatment are required.

## AUTHOR CONTRIBUTIONS

P.S. conceived and designed research; E.R.M.Z, E.C.H., C.H., M.T., W.S.D, M.T., C.L.A., and P.S. performed experiments; E.R.M.Z., C.L.A, RAB, and P.S. analyzed data; E.R.M.Z., C.L.A, and P.S. interpreted results of experiments; E.R.M.Z and C.L.A prepared figures; E.R.M.Z, C.L.A, and P.S. drafted manuscript, E.R.M.Z, E.C.H., W.S.D, R.C.H, M.T., E.C.M., J.P.K, C.L.A, and P.S. edited and revised manuscript; E.R.M.Z, E.C.H., W.S.D, R.C.H, M.T., E.C.M., J.P.K, C.L.A, and P.S. approved final version of manuscript.

## FUNDING INFORMATION

This research was supported in part by National Institute of Health grants P30DK072476 (PS), U54GM104940 (JPK), and T32 AT004094 (ERMZ).

## CONFLICT OF INTEREST STATEMENT

The authors have nothing to disclose.

## ETHICS STATEMENT

The study was conducted in conformity with the Declaration of Helsinki at Pennington Biomedical Research Center after approval from the Institutional Review Board. The study was registered at ClinicalTrials.gov (NCT04530747) prior to enrollment of the first study participant. All patients provided written informed consent.

## Supporting information


Figure S1.
Tables S1–S3.
