# Supplementary material for: Effects of metformin on glucose metabolism and mitochondrial function in patients with obstructive sleep apnea: A pilot randomized trial
Source: Physiol Rep. 2024 Feb 12;12(3):e15948. doi: 10.14814/phy2.15948 (PMC10861357; doi:10.14814/phy2.15948)
Supplement: Supplementary file 1 — Figure S1. Tables S1–S3. [file PHY2-12-e15948-s001.docx]

**Supplementary Tables**

**Table S1.** Effect of metformin treatment on body weight, body composition, habitual dietary intake, sleep and physical activity. HEI: healthy eating index; ESS: Epworth sleepiness scale; PSQI: Pittsburg sleep quality index; IPAQ: International Physical Activity Questionnaire. Data presented are least square means, standard error, and p-value from mixed model analysis.

| Variable | Placebo (n=8) | | | Metformin (n=8) | | | Group Difference | P value  Time*treatment |
| --- | --- | --- | --- | --- | --- | --- | --- | --- |
|  | Baseline | Follow-up | P value | Baseline | Follow-up | P value |  |  |
| Weight (Kg) | 107.9 ± 5.3 | 109.2 ± 5.6 | 0.16 | 111.4 ± 5.3 | 110.8 ± 5.6 | 0.53 | 2.0 ± 1.3 | 0.16 |
| Waist Circumference (cm) | 118.5 ± 5.0 | 119.1 ± 5.4 | 0.63 | 114.3 ± 5.0 | 113.9 ± 5.4 | 0.82 | 1.0 ± 1.9 | 0.62 |
| Hip Circumference (cm) | 116.9 ± 1.8 | 118.0 ± 2.2 | 0.52 | 118.9 ± 1.8 | 119.0 ± 2.2 | 0.97 | 1.0 ± 2.4 | 0.68 |
| Wait to Hip Ratio | 0.99 ± 0.04 | 1.03 ± 0.04 | 0.07 | 0.96 ± 0.04 | 0.96 ± 0.04 | 1.0 | 0.04 ± 0.03 | 0.21 |
| Neck Circumference (cm) | 43.3 ± 1.3 | 43.5 ± 1.2 | 0.62 | 44.1 ± 1.3 | 43.8 ± 1.2 | 0.56 | 0.5 ± 0.5 | 0.45 |
| Fat Mass (Kg) | 46.9 ± 3.2 | 47.4 ± 3.2 | 0.48 | 41.0 ± 3.2 | 40.6 ± 3.2 | 0.61 | 0.9 ± 1.1 | 0.40 |
| Fat Free Mass (Kg) | 60.4 ± 4.2 | 61.2 ± 4.1 | 0.19 | 70.4 ± 4.2 | 69.9 ± 4.1 | 0.43 | 1.4 ± 0.9 | 0.14 |
| % Body Fat | 43.7 ± 2.5 | 43.6 ± 2.4 | 0.86 | 37.0 ± 2.5 | 37.0 ± 2.4 | 0.85 | 0.01 ± 0.6 | 0.99 |
| Daily Calorie Intake | 2164 ± 180 | 1992 ± 194 | 0.36 | 1975 ± 180 | 1955 ± 203 | 0.92 | -152 ± 262 | 0.57 |
| Protein (g) | 102 ± 10 | 92 ± 10 | 0.27 | 101 ± 10 | 108 ± 10 | 0.44 | -17 ± 12 | 0.19 |
| Fat (g) | 107 ± 11 | 97 ± 10 | 0.36 | 89 ± 11 | 90 ± 11 | 0.96 | -11 ± 15 | 0.50 |
| Carbohydrate (g) | 193 ± 32 | 188 ± 31 | 0.80 | 189 ± 32 | 171 ± 31 | 0.40 | 13 ± 29 | 0.66 |
| HEI score (0-100) | 46.5 ± 3.9 | 49.1 ± 3.7 | 0.61 | 51.9 ± 3.9 | 52.7 ± 3.9 | 0.89 | 1.8 ± 7.0 | 0.80 |
| ESS score (0-24) | 9.3 ± 1.9 | 7.4 ± 1.4 | 0.14 | 8.3 ± 2.0 | 6.9 ± 1.5 | 0.28 | -0.5 ± 1.8 | 0.80 |
| PSQI score (0-21) | 7.1 ± 0.7 | 5.7 ± 1.6 | 0.35 | 10.3 ± 0.7 | 7.4 ± 1.6 | 0.05 | 1.5 ± 2.0 | 0.45 |
| IPAQ score  (MET minutes/week) | 7723 ± 2342 | 8666 ± 2779 | 0.80 | 11197 ± 2529 | 3872 ± 3098 | 0.12 | -8269± 5400 | 0.18 |

**Table S2.** Effect of metformin treatment on whole body glucose metabolism. AUC_120_: area under the curve during 120 minutes of oral glucose tolerance test; HOMA-IR: Homeostatic model assessment for insulin resistance. Data presented are least square means, standard error, and p-values from mixed model analysis.

| Characteristics | Placebo | | | Metformin | | | Group Difference | P value  Time*treatment |
| --- | --- | --- | --- | --- | --- | --- | --- | --- |
|  | Baseline | Follow-up | P value | Baseline | Follow-up | P value |  |  |
| Fasting glucose (mg/dl) | 98.6 ± 4.8 | 101.9 ± 5.2 | 0.39 | 97.3 ± 4.8 | 98.1 ± 5.4 | 0.83 | 2.4 ± 5.3 | 0.66 |
| Glucose AUC_120_ (mg/dl) | 19482 ± 1613 | 20181± 1807 | 0.58 | 18831 ± 1613 | 17068 ± 1864 | 0.21 | 2461 ± 1820 | 0.20 |
| Fasting insulin (uU/ml) | 18.6 ± 3.7 | 25.7 ± 4.9 | 0.09 | 17.3 ± 3. 7 | 19.3 ± 5.1 | 0.65 | 5.2 ± 5.7 | 0.38 |
| Insulin AUC_120_ (uU/ml) | 10587 ± 1693 | 14808 ± 2500 | 0.01 | 11980 ± 1693 | 10844 ± 2552 | 0.47 | 5358 ± 2076 | **0.02** |
| Insulin/glucose AUC_120_ ratio | 0.54 ± 0.09 | 0.71 ± 0.09 | 0.01 | 0.65 ± 0.09 | 0.65 ± 0.1 | 0.99 | 0.16 ± 0.08 | 0.07 |
| Matsuda Index | 2.84 ± 0.64 | 2.19 ± 0.74 | 0.35 | 2.96 ± 0.64 | 3.12 ± 0.78 | 0.83 | -0.81 ± 0.98 | 0.43 |
| Insulinogenic Index | 0.93 ± 0.23 | 1.13 ± 0.25 | 0.28 | 1.12 ± 0.23 | 1.34 ± 0.25 | 0.27 | -0.02 ± 0.27 | 0.96 |
| Early phase insulin_0-30_ | 56.9 ± 16.0 | 73.8 ± 12. 5 | 0.19 | 76.4 ± 16.0 | 54.2 ± 13.0 | 0.10 | 39.1 ± 17.7 | **0.04** |
| Early phase glucose_0-30_ | 61.9 ± 7.2 | 66.3 ± 7.2 | 0.46 | 66 ± 7 | 48.0 ± 8 | 0.01 | -22.7 ± 8.5 | **0.02** |
| Disposition index | 2.27 ± 0.59 | 2.25 ± 0.61 | 0.98 | 2.84 ± 0.59 | 3.4 ± 0.64 | 0.36 | -0.57 ± 0.8 | 0.49 |
| HOMA IR | 4.83 ± 1.13 | 6.82 ± 1.48 | 0.08 | 4.14 ± 1.13 | 4.82 ± 1.53 | 0.55 | 1.3 ± 1.52 | 0.41 |
| HbA1C % | 5.48 ± 0.10 | 5.59 ± 0.10 | 0.43 | 5.48 ± 0.10 | 5.43 ± 0.10 | 0.75 | 0.17 ± 0.07 | **0.04** |

**Table S3.** Data presented are least square means, standard error.

| State  (pmol/sec/mg) | Placebo (n=7) | | Metformin (n=4) | |
| --- | --- | --- | --- | --- |
|  | Baseline | Follow-up | Baseline | Follow-up |
| **PM-L** | 7.26 ± 2.33 | 4.52 ± 1.22 | 2.11 ± 0.97 | 5.12 ± 0.89 |
| **PM-P** | 12.99 ± 2.59 | 9.56 ± 2.05 | 2.92 ± 1.80 | 8.47 ± 1.37 |
| **PMG-Pc** | 16.25 ± 2.85 | 14.20 ± 2.48 | 4.46 ± 2.22 | 11.53 ± 1.83 |
| **PMGS-Pc** | 33.20 ± 2.46 | 28.79 ± 4.28 | 14.21 ± 3.91 | 21.33 ± 3.77 |
| **S-E** | 24.17 ± 5.29 | 21.89 ± 2.73 | 8.84 ± 2.94 | 14.49 ± 2.86 |
| **PMG-E** | 13.03 ± 1.97 | 11.84 ± 1.62 | 7.34 ± 2.16 | 9.53 ± 1.67 |
| **PMGS-E** | 37.19 ± 5.91 | 33.73 ± 4.27 | 16.18 ± 4.35 | 24.02 ± 4.51 |
| **CIV-E** | 127.58 ± 18.67 | 79.98 ± 9.37 | 48.09 ± 15.36 | 62.00 ± 11.92 |

**Figure S1.** Representative tracing of SUIT protocol. Representative plot of oxygen consumption (red) and concentration (blue) over the course of substrate, inhibitor, uncoupler protocol in vastus lateralis tissue homogenates. Thom: tissue homogenate, MP: Malate + Pyruvate, D: ADP, G: Glutamate, c: Cytochrome c, S: Succinate, F: FCCP, Rot: Rotenone, Ama: Antimycin A, As/TM: Ascorbate/Tetramethyl-p-phenylenediamine, Azd: Sodium Azide.Green labels indicate substrates, blue indicate uncoupler, red indicats inhibitor.

**
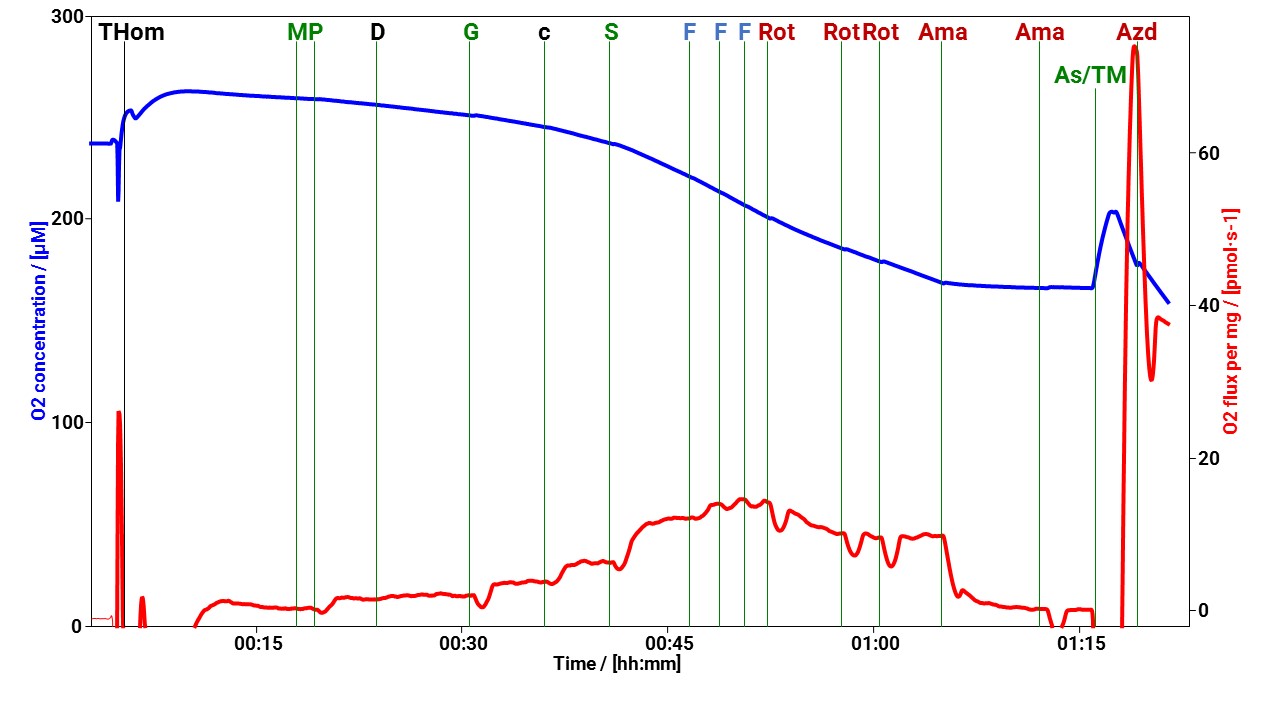
**
